# Short-term and long-term outcomes of single-incision plus one-port laparoscopic surgery for colorectal cancer: a propensity-matched cohort study with conventional laparoscopic surgery

**DOI:** 10.1186/s12876-023-03058-x

**Published:** 2023-11-29

**Authors:** Mingyi Wu, Hao Wang, Xuehua Zhang, Jiaolong Shi, Xiaoliang Lan, Tingyu Mou, Yanan Wang

**Affiliations:** 1 Department of General Surgery & Guangdong Provincial Key Laboratory of Precision Medicine for Gastrointestinal Cancer, Nanfang Hospital, Southern Medical University, 1838, North Guangzhou Avenue, Guangzhou, 510515 China; 2 First Department of Gastrointestinal Surgery, Hainan General Hospital, Hainan Affiliated Hospital of Hainan Medical University, Haikou, 570311 China

**Keywords:** Colorectal cancer, Minimally invasive surgery, Single-incision plus one-port laparoscopic surgery, Conventional laparoscopic surgery, Propensity-score match

## Abstract

**Background:**

Single-incision plus one-port laparoscopic surgery (SILS + 1) has been demonstrated to be minimally invasive while possessing better cosmesis and less pain compared with conventional laparoscopic surgery (CLS). However, SILS + 1 as an alternative to CLS for colorectal cancer is still controversial.

**Methods:**

A total of 1071 patients who underwent curative laparoscopic surgery for colon cancer between 2015 and 2018 were included. Of these patients, 258 SILS + 1 cases and 516 CLS cases were analyzed using propensity score matching. The baseline characteristics, surgical outcomes, pathologic findings and recovery course, morbidity and mortality within postoperative 30 days and 3-year disease-free and overall survival were compared.

**Results:**

Baseline characteristics were balanced between the groups. The mean operating time was significantly shorter in SILS + 1 group, with less estimated blood loss. Tumor size, tumor differentiation, number of harvested lymph nodes, resection margin and pathologic T, N, TNM stage was similar between the groups. There was no significant difference in overall perioperative complications. Uni- and multivariate analyses revealed that SILS + 1 was not a risk factor for complications. Postoperatively, SILS + 1 group showed faster recovery than CLS group in terms of ambulation, bowel function, oral intake and discharge. The 3-year disease-free survival rates of SILS + 1 and CLS groups were 90.1% and 87.3%(p = 0.59), respectively and the 3-year overall survival rates were 93.3% vs. 89.8%(p = 0.172).

**Discussion:**

Our study revealed that SILS + 1 is safe, feasible, oncologically efficient, and may be considered as a surgical option for selected patients with colorectal cancer.

**Supplementary Information:**

The online version contains supplementary material available at 10.1186/s12876-023-03058-x.

## Introduction

Laparoscopic surgery for colorectal cancer has been demonstrated to be safe, minimally beneficial and oncologically efficient compared with open surgery in short-term and long-term outcomes [1–9]. Thus, laparoscopic surgery for colorectal cancer has become an alternative treatment to open surgery around the globe. However, conventional laparoscopic surgery (CLS) would normally require five abdominal incisions for trocars and one mini-laparotomy incision for specimen extraction. Each incision could be associated with pain and wound complication including wound infection and abdominal wall ventral hernia, etc. Hence, in the further pursuit of minimal invasiveness, single-incision laparoscopic surgery (SILS) has been attempted and reported by several surgeons [10–14]. Despite these encouraging results, SILS is a highly technical demanding procedure including collision of instruments motions, poorer surgical field exposure and in-line viewing. As a result, potential disadvantages such as steep learning curve, prolonged operating time and increased surgeon fatigue associated with SILS have become obstacles to the generalization of this technique [15]. Insertion of an additional port to SILS, namely SILS plus one-port laparoscopic surgery (SILS + 1) or reduced-port laparoscopic surgery, was introduced to overcome these technical challenges [16–21]. In our previously reported retro- and prospective studies comparing SILS + 1 with CLS for rectosigmoid cancer, we found that SILS + 1 with only one surgeon and one camera operator was not only man-power conserving, but also safe, feasible and could overcome the limitations of SILS while maintaining the advantages of SILS [22, 23].

The aim of this study was to present our consecutive experience on SILS + 1 for colorectal cancer and determine the short-term and long-term clinical outcomes in comparison with CLS in a propensity score matched manner.

## Materials and methods

### Patients

**Fig. 1:**
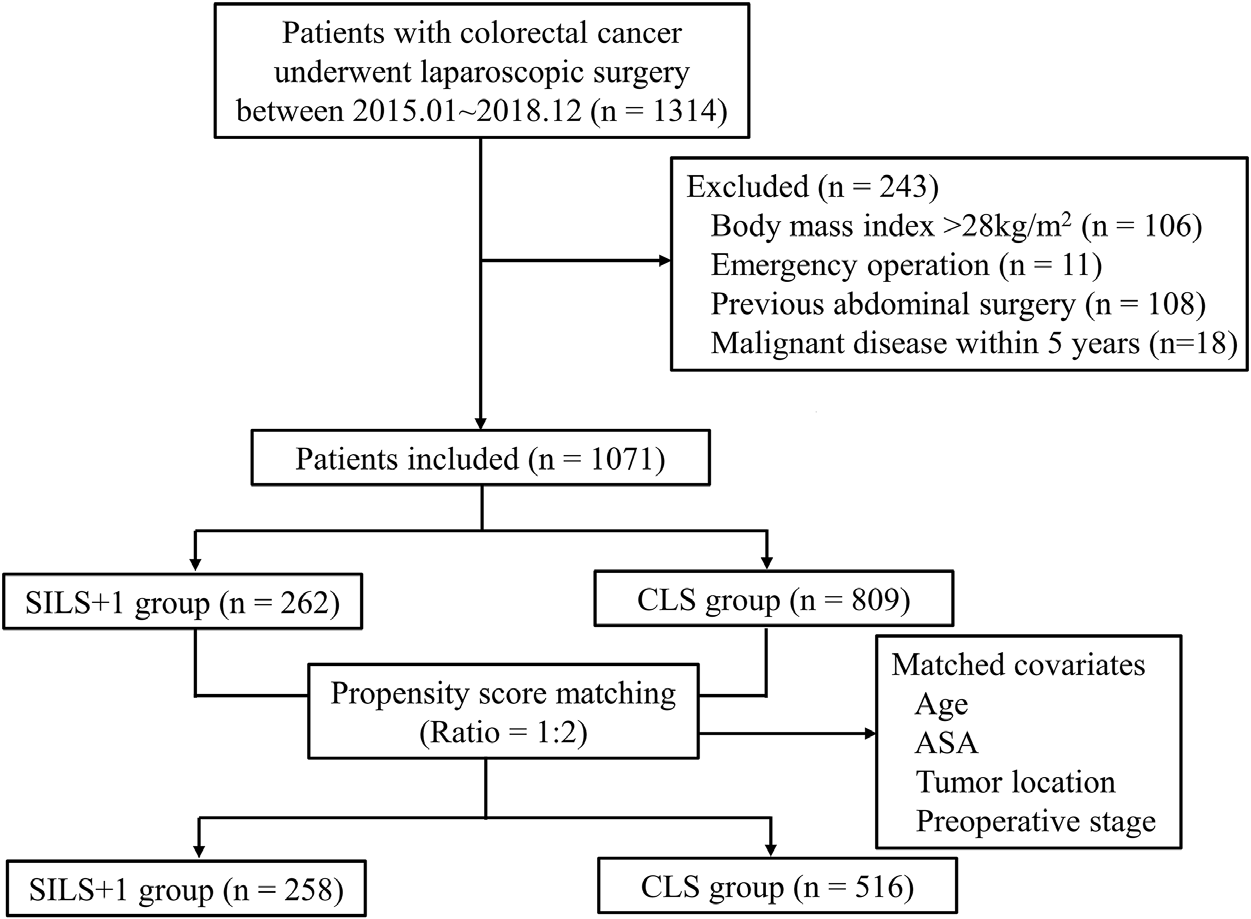
Flow diagram for this study. SILS + 1, single-incision plus one-port laparoscopic surgery; CLS, conventional laparoscopic surgery

We attempted to perform SILS + 1 since 2012 [22], after the learning curve was overcome in 2014 [24]. Between January, 2015 and December 2018, 2257 consecutive colorectal cancer patients received curative laparoscopic surgery (SILS + 1 or CLS) in the Dept. of General Surgery, Nanfang Hospital, Southern Medical University. By selecting the patients according to: (1) 18＜age＜80; (2) primary single tumor diagnosed as adenocarcinoma by endoscopic biopsy (papillary adenocarcinoma, tubular adenocarcinoma, mucinous adenocarcinoma, signet ring cell carcinoma, and poorly differentiated adenocarcinoma); (3) tumor locating at the colon or upper rectum (defined as 10 cm from the anal verge to ileocecal valve; 4) tumor diameter＜6 cm; 5) clinical staging of T1-4N0-2M0 according to the 8th AJCC Cancer Staging Manual; and 6) ASA grading I till III, the clinical data including clinicopathologic characteristics, surgical information, recovery course and morbidities of 1314 patients were drawn from a prospective maintained institutional colorectal cancer database [25]. After excluding 243 patients: (1) body mass index (BMI)＞28.0 kg/m^2^; (2) emergency surgery due to bowel obstruction, perforation or massive hemorrhea; (3) previous abdominal surgery (appendectomy not excluded); (4) malignant diseases within in prior five years, 1071 patients underwent curative laparoscopic surgery were analyzed (262 patients underwent SILS + 1 and 809 patients underwent CLS). Then, the SILS + 1 cases were matched to CLS group using propensity score matching analysis. The matching covariates were: age, ASA grading (I/II/III), tumor location (upper rectum/ sigmoid colon/ left sided colon/ right sided colon), clinical TNM stage (I/II/III) according to the 8th AJCC Cancer Staging Manual. Propensity score matching was applied at a ratio of 1:2 for the SILS + 1 (n = 258) vs. CLS (n = 516) groups (Fig. 1). Baseline characteristics, surgical outcomes, pathologic findings and recovery courses, postoperative morbidity and mortality and 3-year disease-free and overall survival rates were subsequently compared and analyzed between these two groups.

CLS and SILS + 1 surgical informed consents were obtained from all patients with explanation of surgical procedures and risks in details. The ethics committee of Nanfang Hospital approved this study (NFEC-2021-396). All procedures have been performed in accordance with the ethical standards of 1964 Helsinki Declaration and its later amendments.

### Surgical technique

Surgeries were performed by surgeons who had completed over 200 successful CLS cases and 50 SILS + 1 cases after passing the SILS + 1 learning curve, with regard to our previous study [24]. Two surgeons participated in the study.

**Fig. 2:**
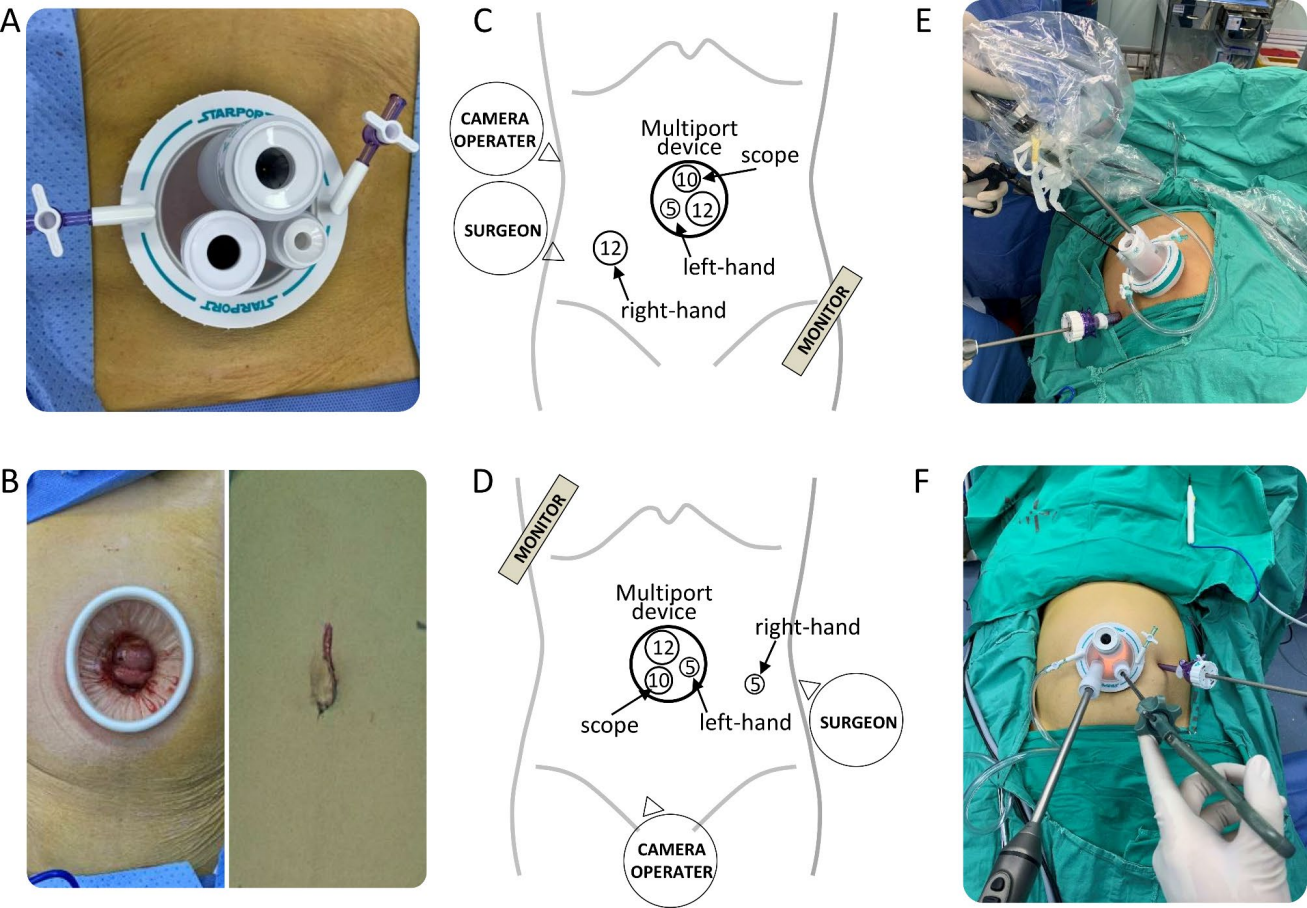
**A**) Multiport device placed at the periumbilical incision. **B**) Periumbilical incision for specimen extraction. **C**) 12-mm trocar placed in the right lower quadrant abdomen for left hemicolectomy or sigmoid-rectal resection. **D**) 5-mm trocar placed at the left quadrant abdomen for right hemicolectomy or transverse colon resection. Laparoscopic instrument placement for **E**) left and **F**) right hemicolectomy with SILS + 1

SILS + 1 and CLS were both performed strictly in accordance with the Japanese Society for Cancer of the Colon and Rectum (JSCCR) guidelines for the treatment of colorectal cancer [26]. Following general anesthesia, patients were placed in the lithotomy position (left hemicolectomy or sigmoid-rectal resection) or in supine position with legs set apart (right hemicolectomy of transverse colon resection). In SILS + 1, a 4- to 5-cm periumbilical incision was made. Then, a multiport device (SURGAID MEDICAL; XIAMEN, CHINA) was placed at the incision (Fig. 2A). Another 12-mm or 5-mm trocar was placed (12-mm trocar placed at the lower right quadrant abdomen for left hemicolectomy or sigmoid-rectal resection (Fig. 2C and E); 5-mm trocar placed at the left quadrant abdomen for right hemicolectomy of transverse colon resection (Fig. 2D F). A standard 30° high-resolute laparoscopy was used through the multiport device. For better exposure, slings of the transverse mesocolon in right- and left-hemicolectomy were applied with purse suturing needles. The procedure consisted of medial-to-lateral laparoscopic mobilization of the segment of the colon or rectum followed by dissection of the bowel together with blood vessels and the accompanying mesecolon/mesorectum and lymph nodes. No prophylactic ileostomy was required in case of rectal cancer surgery. The specimen was extracted via the periumbilical incision (Fig. 2B) and the reconstruction was conducted using an intracorporeal end-to-end anastomosis (left hemicolectomy, sigmoidrectal resection) or extracorporeal side-to-side or end-to-side anastomosis (right hemicolectomy or transverse colon resection).

### Perioperative management

Polyethylene glycol electrolyte solution (1.5 L) was administered the day before surgery for bowel preparation. A single small dose of prophylactic antibiotics (second-generation cephalosporins) was given intravenously 30 min before surgery. Nasogastric tubes were not routinely applied. Postoperative patient-controlled opioid based intravenous analgesia was routinely administered directly after surgery in the recovery room and discontinued on postoperative day (POD) 2. Additional analgesics were allowed in cases of breakthrough pain as recommended by the World Health Organization Analgesic Ladder and at the discretion of the treating ward physician. The drainage tube was not routinely inserted, but mainly determined according to the surgeons’ consideration. If necessary, or in case of rectal cancer cases, the tube was inserted through the additional port in SILS + 1. Drainage tube was removed at the surgeon’s discretion and based on the amount of drainage and the properties of the drained fluid. Patients were discharged when they had full intestinal function recovery, could tolerate a soft diet, no sign of complication and ambulate independently.

### Adjuvant chemotherapy

According to the Japanese Society for Cancer of the Colon and Rectum (JSCCR) guidelines for the treatment of colorectal cancer [26], for postoperative Stage III patients, adjuvant chemotherapy was recommended (either CapOx: oxaliplatin + capecitabine or FOLFOX: fluorouracil + levofolinate calcium + oxaliplatin) for six months. For postoperative Stage II patients, if pMMR with a high risk of recurrence (including T4 disease, poorly differentiated histology, fewer than 12 lymph nodes harvested, and invasion to vascular, lymphatic, or perineural), six months of CapOx of FOLFOX were also recommended. Whereas, for Stage II patients, pMMR without high risk of recurrence, capecitabine monotherapy was recommended for six months. For pStage II patients with dMMR, no adjuvant chemotherapy was recommended.

### Follow-up

All patients were followed up until death or the last follow-up in January, 2022. Follow-up care include: (1) medical history every 3 months for the first 2 years and every 6 months thereafter; (2) physical examination and blood testing with carcinoembryonic antigen and cancer antigen 19 − 9 every 3 months for the first 2 years, and every 6 months thereafter; (3) chest and abdominal computed tomographic scans every 6 months for 3 years; and (4) colonoscopy annually for 3 years. Positron emission tomography–computed tomography was performed if recurrence was suspected.

### Statistical analysis

All statistical analyses were performed using SPSS version 20.0 (SPSS, Inc., Chicago, IL, USA). Descriptive statistics were applied for baseline characteristics analyses. Categorical variables were analyzed by Chi-squared (χ2) test or Fisher’s exact test. For continuous variables, Student’s t test or the Mann-Whitney U test was applied. Logistic regression model was used for uni- and multivariate analyses. Variables were initially entered into the multivariable model based on statistical (p < 0.20) or clinical significance and an “Enter” criterion was used in the final model. Unless otherwise indicated, a two-sided p < 0.05 was considered statistically significant. Survival probability was estimated with the Kaplan-Meier method and compared by log-rank test.

## Results

### Baseline characteristics

The baseline characteristics of the 1071 analyzed patients (262 patients in SILS + 1 group and 809 patients in CLS group, respectively) were shown in Table 1. There was no significant difference observed in age, gender, BMI, ASA score, tumor location and clinical TNM stage between the groups. After propensity score matching, all covariates were balanced and no statistically significant difference were present between SILS + 1 (n = 258) and CLS (n = 516) groups (Table 1).

**Table 1:** Patient baseline characteristics

	All Patients (n = 1071)				Propensity-Matched Patients† (n = 774)		
	SILS + 1 (n = 262) N (%) or mean (SD)	CLS (n = 809) N (%) or mean (SD)	*p*		SILS + 1 (n = 258) N (%) or mean (SD)	CLS (n = 516) N (%) or mean (SD)	*p*
Age (year)	58.32 (11.71)	59.33 (18.33)	0.26		58.35 (11.63)	58.40 (21.29)	0.98
Gender (male)	159 (60.69)	513 (63.41)	0.42		156 (60.5)	325 (63.1)	0.48
BMI (kg/m^2^)	22.09 (2.95)	22.19 (2.77)	0.84		22.10 (2.95)	22.15 (2.78)	0.81
ASA score			0.35				0.84
I	56 (21.4)	173 (21.4)			54 (20.9)	117 (22.7)	
II	191 (72.9)	565 (69.8)			190 (73.6)	373 (72.3)	
III	14 (5.3)	71 (8.8)			14 (5.4)	26 (5.0)	
Primary tumor location			0.73				0.67
Upper rectum	60 (22.9)	125 (15.4)			59 (22.9)	101 (19.6)	
Sigmoid colon	69 (26.3)	318 (39.3)			68 (26.4)	151 (29.3)	
Left sided colon	43 (16.4)	125 (15.5)			42 (16.3)	89 (17.2)	
Right sided colon	90 (26.7)	241 (29.8)			89 (34.5)	175 (33.9)	
Clinical TNM stage‡			0.25				0.82
I	30 (11.5)	122 (15.1)			30 (11.6)	62 (12.0)	
II	150 (57.3)	460 (56.9)			146 (56.6)	280 (54.3)	
III	82 (31.3)	227 (28.1)			82 (31.8)	174 (33.7)	

†Matched covariates including age, ASA score, primary tumor location, and clinical stage (ratio = 1:2)

‡According to 8th edition AJCC staging system

Abbreviation: SILS + 1, single-incision plus one-port laparoscopic surgery; CLS, conventional laparoscopic surgery; BMI, body mass index; ASA, American Society of Anesthesiologists; TNM, tumor-node-metastasis; SD standard deviation

### Surgical outcomes, pathologic findings and postoperative recovery course

Surgical outcomes, pathologic findings and postoperative recovery course are presented in Table 2. During surgery, operating time was significantly shorter in SILS + 1 group. The average difference was approximately 20 min (p < 0.001). Also, less estimated blood loss was observed in SILS + 1 group comparing with CLS group (p = 0.01). There was no significant difference in intraoperative blood transfusion. Due to tumor invasion, adhesion and middle rectal cancer within contracted pelvis, a total of 10 patients (3.8%) required additional trocar in SILS + 1 group during surgery. To be specific, seven patients (2.7%) who required additional one trocar, two patients (0.7%) who required additional two trocars and one patient (0.4%) who required three additional trocars in SILS + 1 group. Though no statistically significant difference was presented in conversion to open surgery, higher rate of conversion to open surgery was observed in CLS group (2.1%) than SILS + 1 group (0.4%) (p = 0.06). After suturing all the incisions, when we summed all the incision lengths including incisions for minilaparotomy and trocar(s), SILS + 1 group had shorter incision length than CLS group (p < 0.01).

**Table 2:** Surgical outcomes, pathologic findings and recovery courses

	SILS + 1 (n = 258) N (%) or mean (SD)	CLS (n = 516) N (%) or mean (SD)	*p*
Operating time (min)	131.80 (43.40)	153.78 (57.39)	< 0.001
Estimated blood loss (ml)	25.45 (29.88)	51.11 (52.16)	0.01
Intraoperative blood transfusion	3 (1.2)	7 (1.4)	0.82
Additional trocar†	10 (3.8)	–	–
Additional 1	7 (2.7)	–	–
Additional 2	2 (0.7)	–	–
Additional 3	1 (0.4)	–	–
Conversion to open surgery	1 (0.4)	11 (2.1)	0.06
Incision length (cm)	6.12 (1.12)	8.23 (2.33)	< 0.01
Tumor size (cm)	2.97 (1.32)	3.01 (1.21)	0.91
Differentiation			0.96
Well	24 (9.3)	68 (13.2)	
Moderate	202 (78.3)	365 (70.7)	
Poor	36 (14.0)	83 (16.1)	
No. of harvested lymph nodes	21.41 (11.12)	23.52 (10.71)	0.41
Proximal resection margin (cm)	9.72 (3.41)	10.40 (3.82)	0.26
Distal resection margin (cm)	6.31 (2.63)	6.62 (2.82)	0.68
Depth of invasion			0.95
T1	23 (8.9)	42 (8.1)	
T2	35 (13.6)	76 (14.7)	
T3	54 (21.0)	122 (23.6)	
T4	135 (52.3)	276 (53.4)	
Lymph node metastasis			0.97
N0	160 (62.0)	338 (66.5)	
N1	74 (28.7)	113 (21.9)	
N2	24 (9.3)	65 (12.6)	
Pathologic TNM stage‡			0.33
I	60 (23.3)	118 (22.9)	
II	98 (38.0)	227 (44.0)	
III	100 (38.8)	171 (33.1)	
Time to ambulation (day)	1.82 (0.9)	2.21 (1.2)	0.02
Time to first flatus (day)	1.77 (0.87)	2.61 (1.07)	< 0.01
Time to first defecation (day)	2.37 (1.32)	3.19 (1.16)	< 0.01
Time to liquid diet (day)	2.11 (0.79)	2.98 (1.29)	< 0.01
Time to soft diet (day)	3.80 (1.18)	4.69 (1.54)	< 0.01
VAS			
POD 1	2.72 (0.54)	2.86 (0.67)	0.48
POD 2	2.50 (0.41)	2.53 (0.61)	0.85
POD 3	2.01 (0.45)	2.28 (0.42)	0.04
POD 4	1.65 (0.61)	1.89 (0.39)	0.02
Discharge day	1.35 (0.49)	1.42 (0.51)	0.76
Postoperative hospital stay (day)	5.23 (2.2)	7.42 (3.6)	< 0.01

†Including patients required one to three additional trocars during operation

‡According to 8th edition AJCC staging system

Abbreviation: SILS + 1, single-incision plus one-port laparoscopic surgery; CLS, conventional laparoscopic surgery; VAS, visual analogue scale; POD, postoperative day; TNM, tumor-node-metastasis; SD, standard deviation

Pathologically, there was no significant difference in tumor size and tumor histological differentiation between the groups. The number of harvested lymph nodes, proximal and distal resection margin was similar between the groups. As for tumor staging, there was no significant difference observed in pathologic T, N and TNM stage (Table 2).

Postoperatively, patients recovered significantly faster in SILS + 1 group in terms of time to ambulation (p = 0.02), time to first flatus (p < 0.01), time to first defecation (p < 0.01), time to tolerate liquid (p < 0.01) or soft diet (p < 0.01), and time to discharge (p < 0.01). The visual analogue scale (VAS) was taken on postoperative day (POD) 1 to 4 and the discharge day in each patient. No significant difference of VAS was observed on POD 1–2 and the day before discharge between the groups (p = 0.48, 0.85 and 0.76, respectively). However, on POD 3–4, VAS was significantly lower in the SILS + 1 group than CLS group (p = 0.04 and 0.02, respectively) (Table 2).

### Morbidity and mortality

Morbidity and mortality are shown in Table 3. The total number of intra- and postoperative complications were similar between the groups (p = 0.09). No patient died in either group. Intraoperatively, three patients (1.2%) in SILS + 1 group experienced intraoperative bleeding due to injury to the Henle’s trunk (two patients) and ileocolic artery (one patient), which required hemo-lock clapping or suture to achieve hemostasis. In CLS group, nine patients (1.7%) experience intraoperative bleeding due to injury to the Henle’s trunk in five patients, ileocolic vein in one patient, inferior mesenteric vein in two patients and middle colic vein in one patient, which required laparoscopic hemostasis with hemo-lock or suture. The intraoperative complication was similar between the groups (p = 0.54).

**Table 3:** Morbidity and mortality

	SILS + 1 (n = 258) N (%)	CLS (n = 516) N (%)	*p*
Total perioperative complication	21 (8.1)	63 (12.2)	0.09
Intraoperative complication†	3 (1.2)	9 (1.7)	0.54
Postoperative complication	18 (7.0)	54 (10.5)	0.12
Wound problem	2 (0.8)	0 (0.0)	0.11
Anastomotic leakage	2 (0.8)	6 (1.2)	0.73
Anastomotic bleeding	3 (1.2)	12 (2.3)	0.41
Lymphorrhagia	2 (0.8)	10 (1.9)	0.36
Intra-abdominal bleeding	0 (0.0)	5 (1.0)	0.18
Intra-abdominal infection	1 (0.4)	3 (0.6)	0.99
Ileus	2 (0.8)	4 (0.8)	0.99
Cardiocerebral events	1 (0.4)	0 (0.0)	0.33
Respiratory infection	1 (0.4)	3 (0.4)	0.99
Uroschesis	4 (1.6)	11 (2.1)	0.78
Mortality	0 (0.0)	0 (0.0)	–
Clavien-Dindo classification			0.45
I	6 (2.3)	21 (4.1)	
II	6 (2.3)	16 (3.1)	
III	6 (2.3)	17 (3.3)	

† Three and nine patients in SILS + 1 and CLS groups respectively experienced intraoperative complication of bleeding

Abbreviation: SILS + 1, single-incision plus one-port laparoscopic surgery; CLS, conventional laparoscopic surgery

There was no significant difference in postoperative complications between the groups (p = 0.12). A total of 18 patients (7.0%) in SILS + 1 group and 54 patients (10.5%) in CLS group experienced postoperative complications. According to the Clavien-Dindo classification, there was no significant difference observed between the groups (p = 0.45). When further analyzing the grade III morbidities: in SILS + 1 group, three patients had anastomotic bleeding after surgery and went through endoscopic hemostasis with clips; two patients with postoperative intra-abdominal infection received ultrasound guided abdominal puncture drainage and one patient with minor acute cardiocerebral events received emergency PCI under local anesthesia. Whereas, in CLS group, six patients suffered from anastomotic leakage, who received re-operation to conduct abdominal washing, drainage and either ileostomy or transverse colostomy; six patients had anastomotic bleeding, and among them, four patients were successfully managed with endoscopic hemostasis, while the other two patients had to experience re-operation; three patients with postoperative intra-abdominal bleeding had re-operation and two patients with postoperative intra-abdominal infection received abdominal puncture drainage.

To analyze the risk factors for morbidity and mortality, uni- and multivariate analyses were conducted (Table 4). Variables were initially entered into the multivariable model based on statistical (p < 0.20) or clinical significance and an “Enter” criterion was used in the final model. Especially, due to its clinical significance, pathologic TNM stage was also included in multivariable model, although its p value was more than 0.20 in univariate analyses. As a result, SILS + 1 as surgical procedure, gender, BMI ≥ 24 kg/m^2^, ASA score, pathologic TNM stage, especially stage II and III were not considered as risk factors for postoperative morbidity and mortality.

**Table 4:** Uni- and multivariate analyses of factors associated with postoperative morbidity and mortality

Factors	Univariate				Multivariate		
	OR^†^	95% CI	*p*		OR^†^	95% CI	*p*
Surgical procedure							
CLS	1				1		
SILS + 1	0.64	0.67–1.12	0.12		0.60	0.34–1.05	0.07
Age							
< 65 years	1						
≥ 65 years	1.25	0.75–2.07	0.39				
Gender							
Female	1				1		
Male	1.65	0.96–2.82	0.07		1.61	0.93–2.78	0.10
BMI (kg/m^2^)							
< 24	1				1		
≥ 24	1.56	0.96–2.55	0.08		1.53	0.93–2.78	0.09
ASA score			0.14				0.08
I	1				1		
II	0.77	0.44–1.37	0.38		0.69	0.37–1.29	0.24
III	1.80	0.70–4.67	0.22		1.74	0.65–4.69	0.27
Primary tumor location			0.63				
Upper rectum	1						
Sigmoid colon	0.70	0.36–1.36	0.29				
Left colon	0.74	0.34–1.59	0.45				
Right colon	0.67	0.35–1.28	0.23				
Tumor size (cm)							
< 4	1						
≥ 4	1.19	0.73–1.96	0.49				
Pathologic TNM stage			0.31				0.16
I	1				1		
II	0.80	0.39–1.62	0.53		0.76	0.36–1.60	0.47
III	1.21	0.60–2.45	0.59		1.30	0.60–2.82	0.51

†OR and corresponding CI were estimated by using logistic regression model

Abbreviation: OR, odds ratio; CI, confident interval; SILS + 1, single-incision plus one-port laparoscopic surgery; CLS, conventional laparoscopic surgery; ASA, American Society of Anesthesiologists; BMI, body mass index; TNM, tumor-node-metastasis

### Oncologic outcomes

**Fig. 3:**
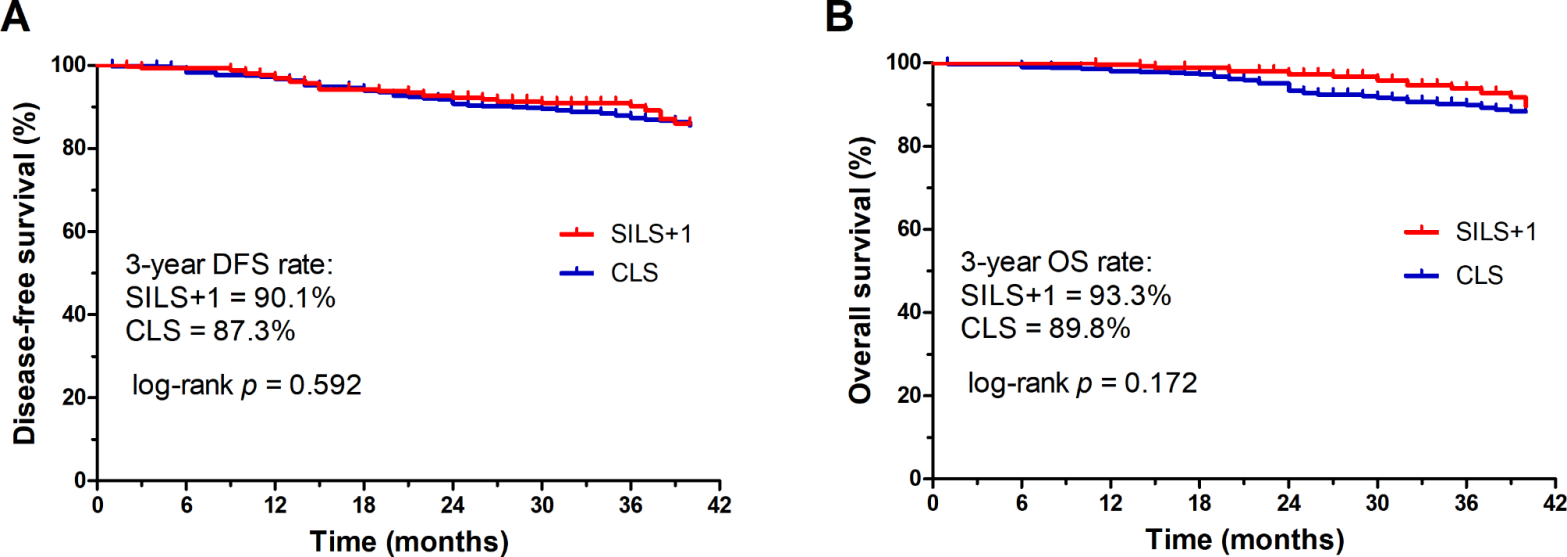
**A**) 3-year disease-free survival. **B**) 3-year overall survival

After surgery, a minimum follow-up of 36 months was required and achieved for each patient in the two groups. The median follow-up period was 46.1 months, with a total of 27 patients (3.5%) lost to follow-up (18 in the SILS + 1 group and 9 in the CLS group). The 3-year disease-free survival rates of SILS + 1 and CLS group were 90.1% and 87.3%, respectively (p = 0.592), and the 3-year overall survival rates were 93.3% and 89.8% (p = 0.172). (Fig. 3). There was no significant difference in 3-year oncologic outcomes between the groups.

## Discussion

During the past decades, large-scale randomized controlled trials have demonstrated the minimal invasiveness and oncological efficacy of laparoscopic surgery for colorectal cancer [1–3, 6, 7, 9, 27]. Based on which, new operative modalities such as SILS and natural orifice transluminal endoscopic surgery (NOTES) have been introduced to minimize the invasiveness. However, SILS and NOTES remained undeveloped, mainly due to the challenging techniques, lack of triangular extraction, overlapping views and requirements of new surgical instruments. Though some surgeons reported satisfactory results of SILS for colorectal cancers, these studies included limited number of cases [11, 28, 29]. Especially, in some studies, authors argued that for distal sigmoid colon cancer or rectal cancer, during SILS anterior resection, it’s difficult to apply linear stapler at the right angle, which may lead to the elevated risk of anastomotic leakage or inadequate distal resection margin [30]. Thus, SILS + 1 with an additional port was attempted to overcome the above obstacles. These authors reported SILS + 1 as a safe and feasible procedure [16, 17, 31].

This study was conducted based on our previous experience on SILS + 1 with one surgeon and one camera operator for rectosigmoid cancers [22, 23]. After the successful application of SILS + 1 for rectosigmoid cancer, we subsequently expanded the indication to all selected colorectal cancers locating from the ileocecal valve to upper rectum. To consolidate the short-term and long-term outcomes of SILS + 1 for colorectal cancer, this comparative study using propensity-score-matching was designed. Our results support that SILS + 1 was equally safe, feasible and oncologically efficient with CLS for the treatment of colorectal cancer. Meanwhile, advantages including reduced operating time, less blood loss, better cosmesis, less pain and faster recovery was demonstrated with SILS + 1.

In this study, the completion rate of SILS + 1 was 95.8%. Among the conversion cases, 10 patients required additional trocar(s) during surgery, with the reason of late staged tumor invading adjacent structures in five patients, two patients had low rectal cancer and narrow pelvis, severe adhesion occurred in the other three patients. Conversion to open surgery occurred in one case due to accidental injury to the Henle’s trunk. The conversion rate of SILS + 1 was comparable to those reported in literatures of 3.5-7.5% [30], and was comparable to 9.8% in our previous prospective study [23]. The high success rate of finishing SILS + 1 could suggest that SILS + 1 for colorectal cancer was feasible in clinical practice. Pathologically, the similarity in number of harvested lymph nodes, proximal and distal resection margins showed that SILS + 1 did not compromise the surgical principles with CLS.

Operating time is another key parameter suggesting the feasibility of a surgical procedure. Though controversy existed in operating time between reduced-port laparoscopic surgeries comparing with CLS, in Lim’s report, operating time was longer in reduced-port group (255.5 min vs. 144.6 min, p＜0.001) [17]. However, in many other studies, authors found that operating time was significantly shorter in reduced-port group [19, 21, 32]. Many author contributed the reduced operating time to the smaller tumor size, relatively earlier tumor stage and difference in surgery types [19, 21]. But in this study, the tumor size, tumor staging and tumor location were balanced through propensity-score-matching, still operating time was significantly shorter in SILS + 1 group. The result was repeated again after our previous findings [22, 23]. The explanation was the hypothesis that SILS + 1 minimized the role of the assistant. Jung speculated that the camera view for the assistant during CLS was mirror image, movements of instruments could sometimes confuse the unskilled assistant, causing involuntary injuries when handling tissues and thus, prolonged operating time [33]. Whereas, in SILS + 1, the procedure was entirely coordinated and conducted by the highly experienced primary surgeon, thus avoiding the dangerous situations caused by unskilled assistants, which would potentially decrease the operating time. The operating time in this study was 131.8 ± 43.4 min in SILS + 1 group, which was similar to other reported reduced-port laparoscopic surgeries [32–34].

Surgical safety, measured mainly by intra- and postoperative complications, was one of the primary concerns of this study. The total complication rate was 8.1% in SILS + 1 group and 12.2% in CLS group, which was comparable to other studies [4, 9, 16, 17, 19, 21, 32, 35]. There was no significant difference observed in either intra- or postoperative complications between the groups. Neither significant difference existed in CD classification categories. When analyzing through uni- and multivariate analyses for risk factors concerning complication, SILS + 1 was not identified as a risk factor. All the above suggested the non-inferiority of SILS + 1 to CLS in surgical safety. Moreover, as we looked into the grade III complication cases, we found that more patients received re-operation in CLS group. The reason might be that SILS + 1 reduced the inflammation and surgical stress response after surgery than CLS. Once complications occurred, patients underwent SILS + 1 might tend to experience milder infection than CLS, which could promote the possibility of successfulness in conservative treatment [23, 36].

During recovery, time to ambulation, first flatus, first defecation, oral intake recovery and hospital stay was significantly shorter in SILS + 1 group in this study, which was repeatedly demonstrated by other reduced-port surgery studies [19–21, 33]. For postoperative pain assessment, when analyzing with VAS, we found that on POD 3 and 4, the VAS was significantly lower in SILS + 1 group, which was also observed in Song’s report and our previous studies [19, 22, 23]. We believed that the three additional 5-mm trocar incisions in CLS group not only augmented the total incision lengths, but also significantly up-regulated the painful feeling when we encouraged the patients to increase ambulation on POD 3–4. Though we used identical recovery pathway for all patients, we thought that the less pain in SILS + 1 group contributed to the earlier ambulation and eventually, resulted in faster recovery of bowel movement.

To ascertain the oncologic efficacy of SILS + 1 compared with CLS for colorectal cancer, in our study, the 3-year disease-free survival rates of SILS + 1 and CLS group were 90.1% and 87.3%, and the 3-year overall survival rates were 93.3% and 89.8% (p = 0.172). Though there were studies focusing on SILS or SILS + 1 laparoscopic surgeries, limited long-term oncologic outcomes were provided [14, 30, 37, 38]. In Kim’s study the 3-year overall and disease-free survival rate for single-incision laparoscopic surgery for sigmoid colon cancer were 94.5% and 89.5% [38]. Also, in Hirano, Y’s analyses on SILS + 1 for rectal cancer, at a median follow-up period of 30 months, 94.3% patients enrolled had no tumor recurrence [30]. Considering that the majority of patients enrolled in our study was stage III tumors (38.8% in SILS + 1 group), and we also included right-sided colon cancer in the analyses, our 3-year oncologic results of SILS + 1 group was comparable to these reports. Meanwhile, the oncologic results of CLS group showed better survival rates than COLOR and COLOR II trials [7, 39], Suggesting that in our study, the oncologic principles for either SILS + 1 or CLS was assured. Since there was no significant difference between the two groups in terms of 3-year oncologic survivals, the comparable oncologic outcomes of SILS + 1 with CLS could be well demonstrated.

Several limitations of our study should be noted: first, although we prospectively collected the data, and conducted propensity-score-matching to minimize the selection bias, and promote the homogeneity of the study, the retrospective nature of the study introduced inherent bias. Second, the study was only carried out in one institution. Additionally, cosmetic outcomes and patient satisfaction of cosmetic effects were not compared in the study, though we believed that the major concern for colorectal cancer patients was not cosmesis. Thus, well-designed multi-center large-scale prospective randomized controlled trials are still needed to further establish the benefits of SILS + 1 in colorectal cancer.

## Conclusion

The present study provides further evidence supporting that SILS + 1 for colorectal cancer is oncologically safe, feasible and would benefit patients in terms of faster recovery, less pain, and may be considered as a surgical option for selected patients with colorectal cancer.

### Electronic supplementary material

Below is the link to the electronic supplementary material.

Supplementary Material 1. Slings of the transverse mesocolon in right-hemicolectomy were applied with purse suturing needles.

Supplementary Material 2. Slings of the transverse mesocolon in left-hemicolectomy were applied with purse suturing needles.

## Data Availability

The datasets used and analysed during the current study are available from the corresponding author on reasonable request.

## Abbreviations

SILS + 1 Single: incision plus one-port laparoscopic surgery
CLS: Conventional laparoscopic surgery
SILS: Single-incision laparoscopic surgery
BMI: Body mass index
JSCCR: Japanese Society for Cancer of the Colon and Rectum
POD: Postoperative day
VAS: Visual analogue scale
CD: Clavien-Dindo
NOTES: Natural orifice transluminal endoscopic surgery
