# Supplementary figures and images for: Short-term and long-term outcomes of single-incision plus one-port laparoscopic surgery for colorectal cancer: a propensity-matched cohort study with conventional laparoscopic surgery
Source: BMC Gastroenterol. 2023 Nov 29;23:420. doi: 10.1186/s12876-023-03058-x (PMC10687908; doi:10.1186/s12876-023-03058-x)

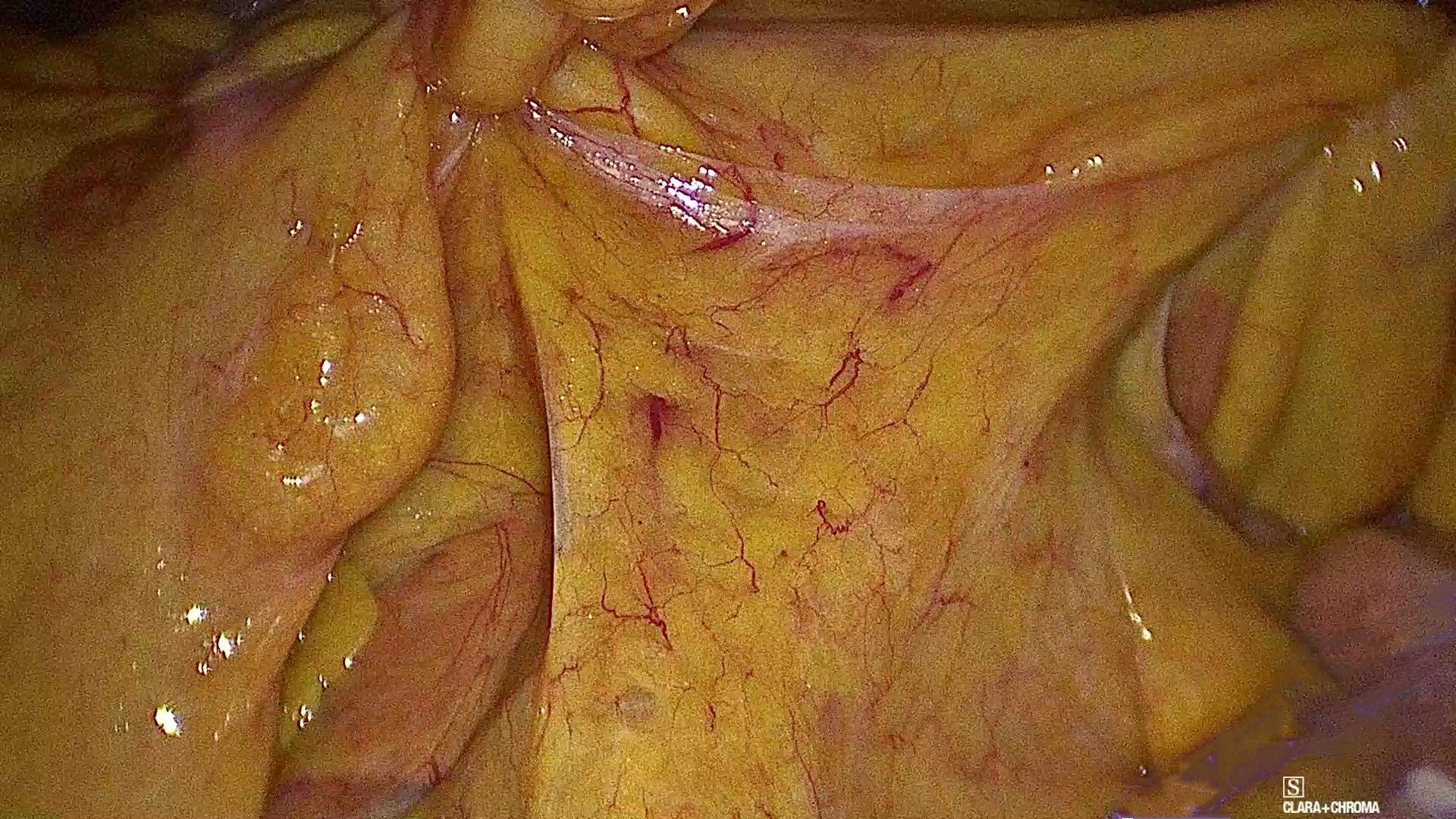

Supplement: Supplementary file 1 — Supplementary Material 1. Slings of the transverse mesocolon in right-hemicolectomy were applied with purse suturing needles. [file 12876_2023_3058_MOESM1_ESM.jpg]

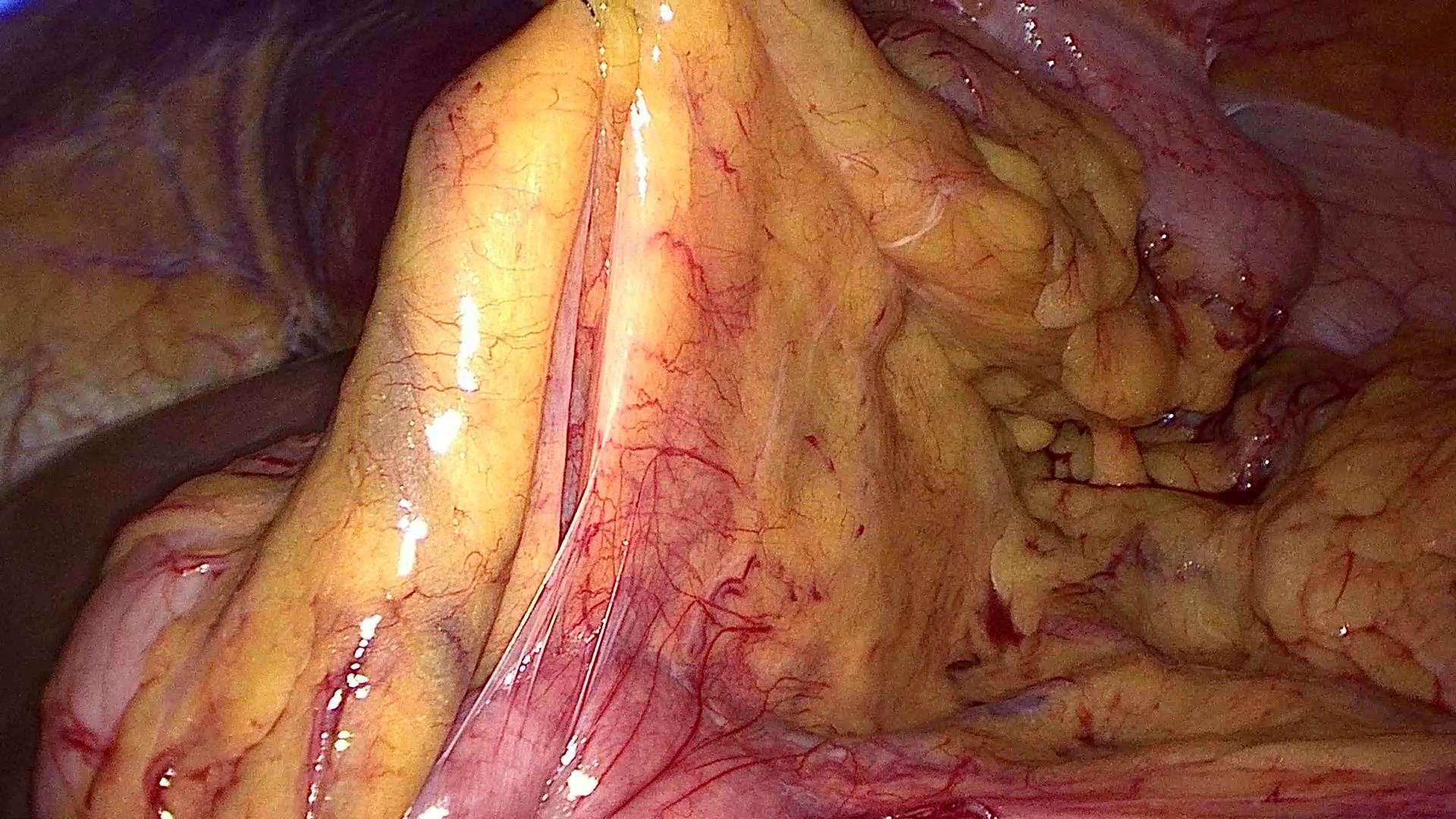

Supplement: Supplementary file 2 — Supplementary Material 2. Slings of the transverse mesocolon in left-hemicolectomy were applied with purse suturing needles. [file 12876_2023_3058_MOESM2_ESM.jpg]
